# Impact of the COVID-19 Pandemic on Atopic Dermatitis Patients

**DOI:** 10.3390/ijerph19031734

**Published:** 2022-02-02

**Authors:** Joanna Sieniawska, Aleksandra Lesiak, Karol Ciążyński, Joanna Narbutt, Magdalena Ciążyńska

**Affiliations:** 1 Department of Dermatology, Pediatric Dermatology and Oncology Clinic, Medical University of Lodz, 91-347 Lodz, Poland; aleksandra.lesiak@umed.lodz.pl (A.L.); joanna.narbutt@umed.lodz.pl (J.N.); ciazynska.magdalena@gmail.com (M.C.); 2 Institute of Applied Computer Science, Lodz University of Technology, 90-924 Lodz, Poland; karol.ciazynski@gmail.com; 3 Department of Proliferative Diseases, Nicolaus Copernicus Multidisciplinary Center for Oncology and Traumatology, 93-513 Lodz, Poland

**Keywords:** atopic dermatitis, COVID-19, coronavirus, quality of life

## Abstract

Atopic dermatitis (AD) can have a significantly negative impact on quality of life (QoL). The impact of coronavirus disease 2019 (COVID-19) on the AD population is not yet well established. The study comprised 195 patients with diagnosed AD who were asked about their cognitive and preventive behaviors regarding COVID-19 and the accessibility of medical support, including online consultations. Moreover, the patients responded to the self-reported Dermatology Life Quality Index (DLQI) and Hospital Anxiety and Depression Scale (HADS). Most of the patients were worried about being infected with COVID-19. Most of the patients believed that people suffering from skin disease were more prone to be infected with COVID-19 compared with the general population. Most the patients negatively assessed the availability of dermatological treatment during the pandemic. Furthermore, 66.1% of the patients declared using telemedicine. Nearly 50% of patients were discontented with telemedicine, and 1/3 of the patients did not mind the use of telemedicine. AD during the COVID-19 pandemic was associated with a lower overall health rating and life satisfaction and impaired QoL related to mental health in a Polish population. These results provide original information that can be applied in dermatologic patient screenings to evaluate the state of depression and anxiety during the epidemic period.

## 1. Introduction

Atopic dermatitis (AD) is a common, chronic, inflammatory and pruritic disease characterized by eczematous skin lesions, skin pain and sleep disturbances [1]. It is a disease of complex etiology. Genetic and environmental factors are involved in the pathogenesis of the disease [2,3,4]. The pathophysiology of AD is also involved in dysfunction of the epidermal barrier [5]. The frequency of appearance of AD is 10–20% of children and 1–3% of adults [6]. AD can have a significantly negative impact on the quality of life (QoL), which, in some cases, can lead to social isolation and psychological problems [7].

Although many studies have demonstrated QoL impairment in clinical patients of AD, the impact of coronavirus disease 2019 (COVID-19) on the AD population is not yet well established [8]. The rapidly expanding COVID-19 pandemic has assaulted all aspects of daily life. Preventive measures, such as social distancing and hand hygienic practices along with patient education in terms of disease prevention, are the greatest priority.

The extraordinary, persistent stress associated with coping with a chronic skin condition and the threat of catching COVID-19 creates additional anxiety for patients. Although SARS-CoV-2 is mainly responsible for respiratory disfunctions, not skin disorders, it has an immense impact on dermatology and dermatological patients. A number of chronic skin diseases may be exacerbated by stress, toxic substances and allergens found in disinfectants and cleansers.

Moderate-to-severe AD can be treated by classical systemic drugs, such as methotrexate or ciclosporin A. Patients suffering from AD also benefit from modern systemic treatments that affect the immune system [1,3,9]. However, the data regarding the safety of biological drugs used in AD should be interpreted with caution, since we do not yet possess data for COVID-19. We present detailed changes of QoL, depression and anxiety in atopic dermatitis patients during the COVID-19 pandemic and explore other factors associated with low QoL in these dermatological patients.

The aim of the study is to determine the impact of a pandemic on the mental state and exacerbation of skin lesions in AD patients using appropriate questionnaires. The study identifies the main mental and physical health problems that affect patients with chronic disease and allow determination of their level of severity. We also assess the range of changes in the patients’ functioning in the community, family relationships and limited access to treatment. Thus far, no similar studies have been conducted. It is not known how a global epidemic may affect the AD population. The obtained data will allow determination of this impact in the studied area and to develop appropriate solutions to the problems faced by patients with AD.

## 2. Material and Methods

### 2.1. Survey Structure

The study involved adult patients diagnosed with AD, confirmed in their medical history by a dermatologist. Subjects were patients of the Department of Dermatology, Pediatric Dermatology and Dermatological Oncology, Medical University of Lodz, Poland and the Dermoklinika Centrum Medyczne. The AD identification criteria used were the UK Working Party criteria. The conducted research was an anonymous, voluntary survey. The patients were required to be able to understand and complete the survey on their own.

**Figure 1:**
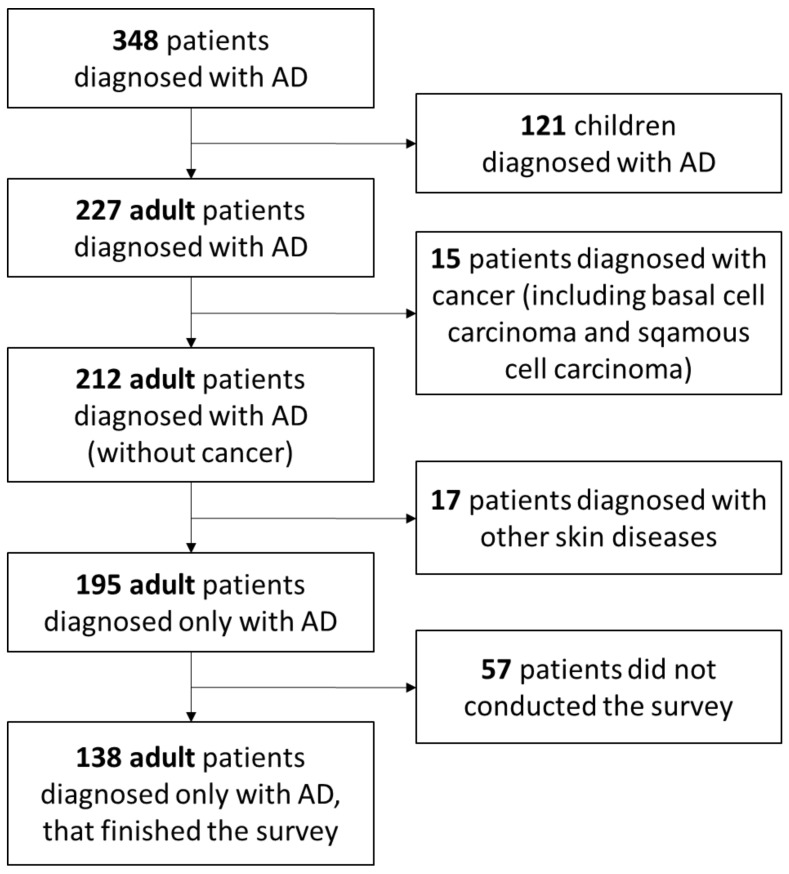
Flow chart of the study inclusion and exclusion criteria.

The patients were informed that completing the survey was tantamount to consenting to the use of data for research purposes while remaining in accordance with the Directive 95/46/EC (General Data Protection Regulation). A survey of patients was conducted in May and August 2020. The study comprised 195 patients with diagnosed atopic dermatitis, of whom 138 (70.8%) completed the examination (Figure 1). The purpose of the examination was explained to the participants, and the strict confidentiality of the participants’ information was maintained.

The study instrument included a designed questionnaire packet with questions about demographic data, including place of residence, gender and source of income. Additionally, information was collected, including The Patient-Oriented SCORing Atopic Dermatitis (PO-SCORAD) and time since diagnosis and treatment. The PO-SCORAD index is a self-assessment score allowing the patient to comprehensively evaluate the actual course of atopic dermatitis (AD), using subjective and objective criteria derived mainly from the SCORAD [10]. Patients were also asked about their cognitive and preventive behaviors regarding COVID-19 and the accessibility of medical support, including online consultation.

Moreover, the patients responded to the self-reported Dermatology Life Quality Index (DLQI) and Hospital Anxiety and Depression Scale (HADS). This research met the ethical guidelines of the institution where the study was performed, including adherence to Polish legal requirements. The procedures of this study complied with the provisions of the Declaration of Helsinki regarding research on human subjects.

### 2.2. Quality of Life

The DLQI was designed to measure the health-related quality of life of adult patients suffering from a skin disease. The DLQI consists of 10 questions concerning the patients’ perception of the impact of skin diseases on different aspects of their health-related quality of life over the last week [11]. The Polish version of DLQI has been applied to measure QoL. Moreover, Szepietowski et al. [12] performed a translation and validation of the Dermatology Life Quality Index (DLQI) and obtained very good results with a Cronbach α efficient value of 0.9.

### 2.3. Depression and Anxiety

The HADS is a self-rating scale that contains two subscales measuring symptoms of depression (HADS-D) and anxiety (HADS-A) during the previous week. It includes seven statements on each disorder, and each response consists of a four-point rating scale (0 to 3), where a higher score depicts a worse condition. For each subscale, the total score is at most 21. A score of ≥11 is considered a clinically significant disorder, whereas a score between 8 and 10 suggests a mild disorder [13].

### 2.4. Analysis

We analyzed both demographic data and the completion percentage by descriptive statistics and the quality of life by the matched paired *t*-test. Both depression and anxiety were analyzed using the Mann–Whitney test for two groups and the Kruskal–Wallis test to evaluate the differences between three or more independent samples. Two-sided p values of 0.05 or less were considered statistically significant. All results were analyzed statistically with Statistica 13.0 (Statsoft, Krakow, Poland).

## 3. Results

One hundred and thirty-eight (138) patients completed the survey questionnaire; 64 (46%) were female and 74 (54%) were male. The patients’ average age was 32.04 ± 7.45 years. The majority of them (74 patients, 53.6%) were living in a large city (over 500,000 inhabitants). Only five patients (3.6%) had relatives or friends diagnosed with COVID-19. No one was asked to quarantine due to suspicion of infection nor had they been infected with COVID-19. The majority of patients (87%) were worried about the economic effects of the pandemic. Most of the patients (69%) were worried about being infected with COVID-19. Moreover, most of the patients (63%) believed that people suffering from skin disease were more prone to be infected with COVID-19 than others.

**Figure 2:**
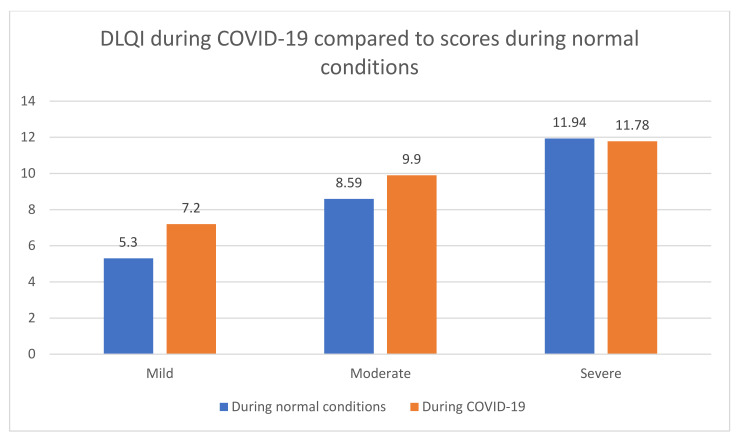
DLQI during COVID-19 compared to the scores during normal conditions.

Study participants with mild and moderate AD had a reduced quality of life. There was no such relationship in the group of patients with severe atopic dermatitis (Figure 2). The DLQI values for mild, moderate and severe AT during normal conditions were 5.3 ± 1.5, 8.59 ± 1.8 and 11.94 ± 2.3, respectively, while, during the COVID-19 pandemic, these were 7.2 ± 1.3, 9.9 ± 1.8 and 11.78 ± 2.1. Similar observations were found for the severity of anxiety and depression. In the group of patients with mild and moderate AD, the severity of anxiety and depression was statistically significant, and we did not observe worsening in patients with severe disease.

Adults with AD during non-epidemic conditions, compared with those with AD during the coronavirus pandemic, had lower average Hospital Anxiety and Depression Scale for anxiety (HADS-A) (8.6 ± 2.1 vs. 7.7 ± 1.8, *p* < 0.05) and depression (HADS-D) (7.9 ± 1.9 vs. 6.0 ± 1.5, *p* < 0.05) scores. Table 1 and Table 2 present the HADS-A and HADS-D scores during normal conditions and during the COVID-19 pandemic [14]. Most of the patients reported exacerbation of skin lesions. The study established that working patients had more severe depression compared with patients who did not work.

More than half of the patients had concerns about the impact of the epidemiological situation on treatment options and negatively assessed the availability of dermatological treatment during the pandemic. We found that 66.1% of the patients declared using telemedicine, and 33.9% of the patients did not use this form of treatment; Table 3. Nearly 50% of patients were discontented with it, and 1/3 of the patients did not mind the use of telemedicine. Furthermore, most patients with moderate to severe atopic dermatitis were not satisfied by the usage of telemedicine; Table 4.

Most patients (60%) who received immunosuppressive and immunomodulating treatments considered changing their treatment. A total of 12% of patients temporarily interrupted their taking of drugs during the pandemic.

## 4. Discussion

The study detailed the exacerbation of skin lesions and the mental state of the patient. There are no studies comparing the scales of DLQI, HADS-A, HADS-D and PO-SCORAD in patients with AD during the epidemic versus before the epidemic. We compared the obtained data with the pre-epidemic study because we did not have a control group [15]. Most of the patients reported exacerbation of the skin lesions localized on the hands and limbs, while the eruption of the face remained stable. Worsening of skin lesions in these areas may be associated with frequent washing of hands and the use of disinfectants, which is a related aspect of the pandemic [16].

Atopic dermatitis is involved with mental health disorders [14,17,18]. The decrease in quality of life and the increase of anxiety and depression more often affects patients with mild and moderate disease. There was not as strong a significant deterioration in the mental health and QoL among patients with severe AD. This may be due to the fact that severe AZS is such a serious disease that other factors, such as a pandemic, are not able to significantly worsen the condition of a patient suffering from the disease already exacerbated almost to its maximum extent.

These data established how serious a disease severe AD is and that even the pandemic and its consequences did not significantly affect the mental state and quality of life of patients with serious forms of AD. The QoL in the groups of patients with mild and moderate AD decreased during the span of the pandemic. What is remarkable is that the QoL of patients with severe AD slightly increased.

The HADS-D and HADS-A in this group both declined. This may derive from the fact that the effects of the outbreak did not influence their daily life. There is also a possibility that the inability for all people to function properly in society made the patients’ difficulties associated with AD less apparent than before the pandemic. As an example, when everybody has to wear a facemask, the skin lesions may be less visible to other people. Furthermore, questions in the DLQI survey relate to the mental state connected with AD. The outbreak may have removed several factors that normally lead to worsening of the general sensation of AD patients

The obtained results were correlated with those in the study conducted before the pandemic by Silverberg et al. and Holm et al. [14,15]. Our study included many other factors related to the functioning of patients in an outbreak. The impact of the pandemic on family relations, access to medical services and use of telemedicine were also assessed. The study showed that *working people had more severe depression*, which was caused by the fear of losing their job.

Moreover, we found that younger people had fewer negative effects of the pandemic compared with older people, because they did not experience any difficulties associated with using public transport. The dependence of daily functioning on factors that were reduced during the epidemic had a negative effect on the mental sphere of patients. Over half of the patients had concerns about the impact of the epidemiological situation on treatment options and negatively assessed the availability of dermatological treatment during the pandemic.

Most of the patients declared using telemedicine, but nearly 50% of them were discontented. This may be related to the fact that telemedicine was not a popular form of contacting a doctor; however, during the pandemic, it became the first method of choice, regardless of the patient’s preferences. Most patients with moderate to severe atopic dermatitis were not satisfied by the use of telemedicine. Thus far, there are no studies with which we could compare our observations. The data obtained by us require further investigation but confirm the dynamic development of this form of medicine and the growing interest on the part of the patients and practical use.

However, in all groups, an improvement in family relations was observed. Isolation caused the necessity of limiting social contacts, which were restricted to close family. A positive effect of pandemic on family relations was observed in all patient groups.

Although, to the best of our knowledge, worsening of the clinical course of pre-existing skin diseases because of COVID-19 infection has not thus far been reported, most of the patients were convinced that AD is a disease that increases the risk of COVID infection. This is related to the chronic course of this disease and the need for treatment with various methods, including immunosuppressants [19].

In addition, some therapies involve the functioning of stationary dermatological units, such as phototherapy [20]. Most of the therapeutic options used to treat more severe forms of AD undoubtedly have an effect on the patients’ immune systems. Both patients and doctors must consider how to choose the appropriate form of treatment in the time of a pandemic so as to not interrupt it and not to cause exacerbation. At the same time, the treatment should be optimal in terms of effectiveness and safety as well as availability to the patient.

Most of the patients treated with immunosuppressants considered changing their treatment. Some patients contacted their doctor regarding this issue. Some patients temporarily interrupted taking the drug, mainly due to the fear of risk involved in transport to the center and possible infection (contact with a doctor and other patients).

Even before the COVID-19 pandemic, it was reported that concerns about biological treatment discontinuation created uncertainty and insecurity and resulted in fear and negative beliefs about the future [21]. A literature review examining biologic therapy compliance and persistence in chronic inflammatory diseases showed that irregular use was associated with lower clinical effectiveness [22]. Meanwhile, maximal improvement of the disease severity is important to reach a normal QoL in psoriatic patients [23].

However, most European and American recommendations agree in stopping treatment of patients on systemic immunosuppressive agents who have tested positive for COVID-19 or who exhibit signs/symptoms of COVID-19 by discontinuing or postponing the systemic immunosuppressive agents until the patient recovers from COVID-19 [24].

The method of the study has certain limitations. Considering that the data for the study was collected via a survey, there is the potential for the results to vary slightly from the truth, considering possible patient bias and the possibility that not all the questions were understood correctly. Furthermore, the study was conducted near the beginning of the outbreak and may require further research, as the patient conditions and QoL may have changed over the course of the pandemic. On the other hand, the study survey was conducted on a large group of patients, it was quick and accessible, was not burdensome for patients and was safe during the pandemic.

## 5. Conclusions

In conclusion, these data support the heavy burden that moderate and mild AD placed on patients during the COVID-19 pandemic. During this extraordinary time, the dermatology community faces unprecedented challenges to improve the mental health and quality of life for people with chronic disease, such as AD. Atopic dermatitis during the COVID-19 pandemic was associated with a lower overall health rating and life satisfaction and impaired QoL related to mental health in the Polish population.

These results provide original information that can be applied in dermatologic patient screenings, as it is important to evaluate the state of depression and anxiety during the epidemic period. The identification of patients who are depressed and would benefit from further support is necessary in order to make individual interventions in time.

The dermatology community should consider that methods, such as multidisciplinary educational programs, psychologic interventions, support to promote behavioral changes, religious/spiritual well-being, music and diet—all of which have been reported to improve the QoL of patients with skin disease—may be especially important during quarantine and self-isolation. The special role of telemedicine in treating patients during epidemics should be emphasized. Due to technological progress and the pandemic, telemedicine has become a new, universal therapeutic option.

The gradual improvements of this form of contact with patients and its widespread use may contribute to increasing the level of patient satisfaction and increasing the effectiveness of treatment. In the group of patients with severe AD, telemedicine may not be sufficient as patients with severe disease require personal medical examinations and forms of treatment. The reasonable use of telemedicine allows for quick access to a specialist and appropriate treatment or qualification for a stationary visit, which improves the treatment process and is beneficial for the patient and the doctor.

Almost 40% of patients declared their willingness to participate in educational support programs, psychologic interventions and counselling to promote behavioral changes and diet. These activities can improve the QoL of patients with skin disease and may be especially important during these extraordinary times, including quarantine and self-isolation. Therefore, preventive and treatment measures are very important for QoL improvement during the COVID-19 pandemic.

## Figures and Tables

**Table 1:** Diagnosis of anxiety during COVID-19 compared to the scores during normal conditions.

PO-SCORAD	HADS-A Score during Normal Conditions [14]	HADS-A Score during the COVID Pandemic	*p*
Mild	6.1 (5.5–6.7)	7.3 (6.0–7.9)	*p* < 0.05
Moderate	9.2 (8.2–10.2)	10.1 (8.6–12.0)	*p* < 0.05
Severe	11.2 (9.2–13.1)	11.3 (9.3–13.0)	*p* = 0.097

**Table 2:** Diagnosis of depression during COVID-19 compared to the scores during normal conditions.

PO-SCORAD	HADS-D Score during Normal Conditions [14]	HADS-D Score during the COVID Pandemic	*p*
Mild	4.6 (4.1–5.2)	5.7 (4.9–6.9)	*p* < 0.05
Moderate	7.4 (6.6–8.2)	8.5 (7.6–10.0)	*p* < 0.05
Severe	9.1 (7.8–10.5)	8.7 (8.3–11.2)	*p* = 0.142

**Table 3:** Usage of telemedicine during COVID-19.

PO-SCORAD	Usage of Telemedicine Yes	Usage of Telemedicine No
Mild	51%	49%
Moderate	42%	58%
Severe	26%	74%

**Table 4:** Level of content within patients using telemedicine during the COVID pandemic.

PO-SCORAD	Level of Content (LoC) Definitely Yes	LoC Yes	LoC Neutral	LoC No	LoC Definitely No
Mild	28%	25%	24%	14%	9%
Moderate	10%	14%	30%	19%	27%
Severe	2%	11%	28%	24%	35%

## Data Availability

The data that support the findings of this study are available from the corresponding author upon reasonable request.
